# Quality Assurance for Clinical Trials

**DOI:** 10.3389/fonc.2013.00311

**Published:** 2013-12-19

**Authors:** Geoffrey S. Ibbott, Annette Haworth, David S. Followill

**Affiliations:** ^1^Department of Radiation Physics, The University of Texas MD Anderson Cancer Center, Houston, TX, USA; ^2^Peter MacCallum Cancer Centre, Melbourne, VIC, Australia; ^3^Radiological Physics Center, Houston, TX, USA

**Keywords:** quality assurance, health care, clinical trials as topic, anthropomorphic phantoms, credentialing, radiation therapy

## Abstract

Cooperative groups, of which the Radiation Therapy Oncology Group is one example, conduct national clinical trials that often involve the use of radiation therapy. In preparation for such a trial, the cooperative group prepares a protocol to define the goals of the trial, the rationale for its design, and the details of the treatment procedure to be followed. The Radiological Physics Center (RPC) is one of several quality assurance (QA) offices that is charged with assuring that participating institutions deliver doses that are clinically consistent and comparable. The RPC does this by conducting a variety of independent audits and credentialing processes. The RPC has compiled data showing that credentialing can help institutions comply with the requirements of a cooperative group clinical protocol. Phantom irradiations have been demonstrated to exercise an institution’s procedures for planning and delivering advanced external beam techniques (1–3). Similarly, RPC data indicate that a rapid review of patient treatment records or planning procedures can improve compliance with clinical trials (4). The experiences of the RPC are presented as examples of the contributions that a national clinical trials QA center can make to cooperative group trials. These experiences illustrate the critical need for comprehensive QA to assure that clinical trials are successful and cost-effective. The RPC is supported by grants CA 10953 and CA 81647 from the National Cancer Institute, NIH, DHHS.

## Introduction

In recent years the approach to treating cancer patients with radiation therapy has evolved considerably. In past decades, it was common for clinicians to base treatment decisions on their individual past experiences, but today it is more common to employ “evidence-based medicine” and derive prescriptions from data obtained through clinical trials (5). The highest-level evidence for any type of therapy originates from systematic reviews of randomized clinical trials.

This article will review the clinical trials programs in the US and elsewhere, and will discuss requirements for quality assurance (QA) procedures specific to participation in these multi-center clinical trials. For related information, the reader is referred to previous publications on the topic (6, 7).

## Clinical Trials

According to data from the Radiological Physics Center (RPC), nearly 70% of all US radiation therapy centers participate to some degree in cooperative group clinical trials (8). About 25% of the centers collectively enroll more than 1,000 patients per year on protocols managed by one or more of the clinical trials groups. For a treatment center to join and continue membership in the cooperative groups, the institution’s physics staff must perform specific QA procedures. For protocols that require or permit radiation therapy, there are particular radiation therapy QA and data submission requirements for each patient entered into the trial. Participation in certain advanced technology protocols such as those requiring three-dimensional (3-D) conformal treatment, intensity-modulated radiation therapy (IMRT), volumetric modulated arc therapy (VMAT), stereotactic radiation therapy, or brachytherapy require a substantial physics effort to qualify the institution to enter patients. Further physics effort often is necessary to submit the required data for each patient treated under protocol.

A clinical trial typically compares a new treatment, or new treatment approach, with the “standard treatment.” In the case of radiation therapy, the standard treatment typically is defined as the most-commonly delivered dose prescription using the most-popular treatment approach.

Clinical trials are classified as Phase I, II, or III to describe the goals of the trial and the level of evaluation that is being performed. Phase I trials generally recruit small numbers of patients and may determine the dose that can be tolerated, the delivery method, or perhaps a novel fractionation procedure. Patients recruited to Phase I trials may be chosen from a select group such as those not expected to respond well to conventional treatments. If the Phase I trial is successful, a Phase II trial will follow and may recruit a larger number of patients. The results from a Phase II study will provide a measure of the effectiveness of the new treatment in a group of patients where it is thought the new treatment may be superior in outcome to the standard treatment. This can help determine the sample size required for a definitive trial by providing an estimate of the variability in response, an estimate of the difference in response expected between the new treatment and the conventional treatment, and identification of sub-populations that respond differently to the larger patient group. It may take many years to complete Phase I and II trials; on occasion these trials are combined.

A trial that randomizes patients between two (or more) arms comparing a new treatment against a standard treatment is called a Phase III trial. Phase III trials usually follow smaller Phase I and II trials that test the safety and effectiveness of the new treatment approach. Phase III trials randomize patients to the arms of the study to avoid a systematic difference (or bias) between the groups due to factors other than the new treatment approach. Patients may be further sub-divided, or stratified, if there are other known factors that may influence the patient outcome, such as smoking history. Stratification may reduce the possibility of a trial reporting a null hypothesis (i.e., there is no difference between treatments) when in fact there is a difference (9). Stratification may also be useful for pooling data with other clinical trials to conduct meta-analyses. A single clinical trial may be powered sufficiently to answer only the primary trial question but when multiple studies are combined, there opens the possibility of generating conclusive results for sub-studies and secondary trial questions (10).

A multi-institutional Phase III clinical trial has the potential to set a new standard for clinical management of cancer patients, and the results of such research could therefore be implemented widely. For the results of a clinical trial to be replicated on a national scale, the clinical trial must clearly define the treatment parameters used. In the case of trials involving radiation oncology there are two general approaches:

- When the radiation therapy is not part of the trial question, e.g., in the testing of a new drug, centers are instructed to deliver the radiation therapy using their “standard” approach, even though this might be quite different among centers. Should such a trial be successful it can then be concluded that the study drug will (or will not) result in a superior outcome regardless of the radiation therapy technique.
- A second approach is to define in precise detail how the radiotherapy treatment will be delivered. A strict QA program is necessary to confirm that the dose was delivered according to the protocol. This approach is required for trials that include a question related to the radiotherapy treatment and is becoming more common as potentially a smaller number of patients are required for any trial (11), and hence statistically significant results can be achieved in a shorter data collection time. A demanding QA program is particularly relevant when the expected difference between the arms of the trial is expected to be small.

## Radiation Therapy Technique

The Section “Radiation Therapy Technique” of a trial protocol must provide sufficient detail for planning and treatment delivery to be consistent across participating centers. The level of detail may vary widely across trials depending on many factors relating to the relevance of the radiotherapy treatment to the question the trial aims to answer. Using the example described above, a trial investigating the use of a new drug to delay the onset of disease progression may state only the minimum dose that should be delivered to the PTV and each participating center may then follow their own in-house treatment protocol. A trial comparing a high dose hypofractionated treatment to the lung using non-coplanar, motion compensated fields against a standard treatment schedule using conventional coplanar fields without motion compensation requires a large degree of detail so that the distinction between the two radiotherapy techniques is clear; so that patients do not come to harm due to lack of understanding of the trial protocol, and because the investigational arm may involve centers developing many new techniques to comply with the protocol, each carrying their own risk of misinterpretation due to limited experience.

An example of the contents of the Section “Radiation Therapy Technique” of a trial protocol is shown in Table 1. The Section “Radiation Therapy Technique” should start by stating the planning objectives (e.g., radical or palliative intent), treatment modalities allowed, dose prescription (including details of how and where the dose should be prescribed) and the treatment schedule, including dose per fraction and overall treatment time. Although specifying dose prescription may sound simple, some thought should be put into how the prescription may be interpreted for different planning techniques. For example a trial permitting only conformal radiotherapy using photons may choose to use the recommendations of ICRU 50 and 62 (12, 13) with a prescription based on a point within the center of the treatment volume. A trial that allows only an IMRT approach may use the recommendations of ICRU 83 (14) with dose prescription based on a median dose and a number of planning objectives. Great care must be taken in defining the method of dose prescribing, as the outcome from plans that are produced by the participating centers may vary sufficiently that trial results cannot be interpreted (15, 16). As a general recommendation, the principal investigator (PI) must specify the treatment planning objectives and provide details on how these objectives may be realized with the various treatment planning techniques permitted. For example, a trial that is investigating the influence on tumor control of the timing of radiotherapy relative to surgery may permit 3DCRT and IMRT, with the purpose of IMRT in this scenario to reduce side effects of treatment. As the study endpoint is tumor control due to the timing of surgery, it would not be appropriate in this trial to use IMRT to boost target sub-volumes and hence, in this example the planning objectives would be to deliver a uniform equivalent dose to the treatment volume regardless of treatment planning technique. This may be achieved through specifying the maximum and minimum dose that would be common to 3DCRT and IMRT techniques. The prescribed dose, however, must be chosen so that it is interpreted equivalently by either technique. For example, the same dose value may be prescribed to the ICRU 50 point for the 3DCRT plans and to the median PTV dose for IMRT plans (14, 15). Consequently, dose-volume histograms are often used prospectively to define the intended dose to the target and organs at risk.

**Table 1:** **Example of the table of contents for the Section “Radiation Therapy Technique” of a clinical trial**.

6.0	Radiation therapy
6.1	Dose specification
6.2	Technical factors
6.3	Localization, simulation, and immobilization
6.4	Treatment planning/target volumes
6.5	Clinical structures
6.6	Documentation requirements
6.7	Compliance criteria
6.8	Radiation therapy quality assurance review
6.9	Radiation therapy adverse events
6.10	Radiation therapy adverse events reporting

Details to be provided in the treatment planning section are to be described in a forthcoming report from AAPM Task Group 113. The trial protocol should, for example, state that calculations should be carried out with a heterogeneity correction (where appropriate) and in some cases it may be necessary to specify particular algorithms that may (or may not) be used in a trial. For example, trials involving highly conformal treatment delivery to the thoracic region may insist that the TPS shall utilize a 3D dose calculation algorithm capable of performing calculations which account for variations in lateral scatter in the presence of 3D computed tomography (CT)-defined heterogeneities. Pencil beam algorithms for example, may be excluded for such trials because their performance is known to be inferior in the presence of low-density heterogeneities (17).

Clinical structure definitions may be related to published data relating the dose-planning constraints to clinical toxicities. For example, the QUANTEC papers (18) provide evidence for the dose response in a number of critical organs.

The Documentation Requirements section of the clinical trial protocol (see Table 1) should provide details of the data that shall be submitted for review. Data generally are submitted in electronic format and must be anonymized prior to submission. The data selected for review must be sufficient to confirm that the center has created a treatment plan that conforms to the trial protocol. In most cases, the treatment plan will be submitted in a common format such as DICOM-RT (19) that can read by the cooperative clinical trials group (CCTG) plan review software. In addition the trial may require verification images be submitted along with an end of treatment report to confirm the patient was treated as planned. Additional data may be requested to support future analyses appropriate to the trial end points.

## Role of Medical Physics in Clinical Trials

Multi-center clinical trials operated through CCTGs such as the Radiation Therapy Oncology Group (RTOG), European Organization for Research and Treatment of Cancer Radiation Oncology Group (EORTC-ROG), Trans-Tasman Radiation Oncology Group (TROG), etc., generally have the greatest impact on changing clinical practice as these groups set high standards for trial design, QA, and management, resulting in high quality data to support trial results that can be reproduced in the clinic. Although the process of developing a clinical trial may vary among the groups, the same general principles apply which in turn provides an opportunity for clinical trial groups to collaborate to increase patient recruitment.

A clinical trial typically starts as a small pilot study (which may be conducted in a single center or a small number of centers) or a Phase I and/or II trial to confirm the new treatment is worthy of further investigation. The PIs will be invited by the CCTG to put forward their ideas at the group’s research strategy committee meeting where they can be subject to peer review. Typically peer review will involve a multi-disciplinary audience and usually includes a consumer representative, statisticians, and experienced trial investigators. Following peer review and general agreement from members of the CCTG, the investigator will carry out a feasibility survey to confirm that sufficient numbers of patients can be recruited in a reasonable time frame. At this stage the PI will confirm details of the “standard treatment” and the ability of centers to offer the investigational treatment. Once the feasibility is confirmed, protocol writing and applications for funding can begin.

In writing a protocol, each contributing author is required to follow the standard format relevant to the particular CCTG. Additional sections can be added as required for a particular trial. It would be expected that a medical physicist, or a team of physicists, be involved in the writing of the technical sections of the protocol, including the QA section. This highlights one of the most important roles of the medical physicist in clinical trial involvement. A large responsibility lies with the medical physicist in identifying the level of detail that needs to be included in the protocol and providing instructions that, when appropriate, are not hardware specific and can be followed in the multi-vendor environment commonly associated with CCTG multi-institutional trials.

A medical physicist who has an understanding of the needs of a clinical trial will be able to identify key risk areas that could adversely affect the success of the trial. For example, a physicist can define the variability in the way a particular cancer might be treated, such as cancers in the thoracic region where a variety of techniques for managing tumor motion may lead to a range of outcomes, even if all sites apply the same treatment margins. The medical physicist may play a key role in defining the need for a QA program by defining the risks associated with predicted intercenter variability in treatment delivery (20) and also provide an estimate of the cost for executing the QA program which is required at the time of applying for grants to manage the clinical trial. In addition, the medical physicist may play a role in the risk management process associated with the trial such as by providing guidance on estimating radiation doses due to supplemental imaging techniques. This may vary based on the techniques or vendor specific equipment used. Cone beam CT (CBCT) is a good example where local ethics committees may not approve daily imaging unless good justification for the additional dose can be provided. These key points should be discussed in detail with the trial investigator early in the development of the trial protocol and well before applications for trial funding are made.

Most CCTGs now recognize the need to include a physicist in the trial management committee and in most cases, such a role will be recognized in the author list when the trial results are published. In addition, a medical physicist may act in an advisory role to the CCTG. Prior to opening to patient recruitment, all CCTGs require trial protocols to be approved by a multi-disciplinary committee to ensure that the protocol meets the high standards expected of the trial group. Such committees contain technical representatives, including medical physicists who are typically practicing clinical medical physicists, or physicists that have substantial clinical medical physics experience.

Hospital participation in clinical trials varies; however, it has been shown that patients treated at hospitals that contribute larger numbers of patients to trials do better than those with low contribution rates (21). The clinical medical physicist involvement in clinical trial work may therefore vary, but the importance of this work cannot be understated as the outcome of a clinical trial may affect the way many patients, locally, nationally, and on the international scale, are treated. If asked to assist with clinical trial participation, the medical physicist should attempt to understand the goals of the clinical trial and if necessary, contact the trial physicist or appropriate QA office to confirm the requirements of the trial. The local medical physicist should also carefully read the Radiotherapy and QA sections of the protocol and the requirements for data submission. In some situations additional resources may be required and this should be identified early. Some trials may require modifications to the protocols used locally for patient treatment and ideally a member of staff should be allocated the task of identifying the special needs of a trial to minimize the risk of protocol violations. Investing time in identify the needs of a trial and setting up processes to comply with the protocol early will save time in the long run by avoiding the need to re-submit data.

## Development of a Quality Assurance Program

In the context of a clinical trial, QA involves many different disciplines and a range of tasks including verification of patient eligibility to enter the trial, assurance that all tests and tumor response/toxicity measures are carried out at the time intervals specified in the protocol, verification that data entered into case report forms (CRFs) are accurate and have been entered into the trial database correctly and, finally, assurance that the treatment was delivered according to the trial protocol. In brief, the role of a QA program within a clinical trial can be simply defined as a program to minimize the possibility of systematic discrepancies in treatment management among participating institutions. The specific role of a clinical trials QA program has been described by van Tienhoven et al. who wrote that a trial-specific QA program should include procedures that can be conducted readily, should be able to identify and quantify variations in relevant parameters, should be able to detect and correct significant variations, and should be able to demonstrate an effect on the outcome of the trial (22).

The importance of a good quality control program within a clinical trial has been demonstrated on many occasions (23–25). Pettersen et al. demonstrated the effect on sample size required for a clinical trial in the presence of dose uncertainties, and in turn, Bentzen et al. described the potential clinical effects of these uncertainties (11, 20). Ohri et al. reported a meta-analysis of several trials and demonstrated that in most cases there were correlations between the quality of radiation treatment delivery and patient outcome (26). Fairchild et al. similarly reported a literature review of 17 multicenter clinical trials demonstrating a correlation between radiation therapy quality and trial outcome (27). Insufficient QA in clinical trials, as well as QA that is performed after accrual to the trial has concluded, also have been found to degrade the outcome of a clinical trial. In a clinical trial investigating the use of a hypoxic cytotoxin agent in the treatment of head-and-neck cancers, the impact of non-conformance to the trial protocol was found to be larger than the impact of the trial drug with the data from the non-protocol compliant treatment plans potentially detracting from the ability of the trial to demonstrate the benefits of the drug (21, 28).

The topic of QA in clinical trials is well covered in the forthcoming AAPM publication from Task Group 113. In addition, AAPM Report 86 (7) provides an overview of QA in clinical trials with a focus on the role of various QA groups within the National Cancer Institute in Northern America. Purdy (29) also provides details of the US and European clinical trial programs.

The effort required to conduct a comprehensive QA program has been documented thoroughly. Pawlicki et al. (30) have proposed a risk-based approach to QA similar to those used in industry. The principle to be followed states that the frequency and intensity of verifying a specific function or activity should increase when: (a) the probability that something will go wrong is increased, (b) the degree of damage (e.g., incorrect treatment, treatment of incorrect patient, etc.) that can be caused is increased, or (c) the likelihood that a failure could escape detection is increased. It must be understood that patient safety remains the responsibility of the local hospital, and not the clinical trials group. The role of a clinical trial QA program is therefore not to specify what and how often a particular component should be tested, except to confirm that the local QA program meets national standards. Instead the clinical trial QA program will use a risk-based approach to identify what aspects of the treatment delivery chain are likely to affect the ability to answer the trial question. The QA program must be able to operate within a reasonable budget, have access to appropriately qualified personnel with adequate experience in the appropriate field, not place too great a burden on the treating center so that it prohibits their participation, and set tolerance limits that are appropriate for the accuracy required within the specific trial. The latter point is the most challenging aspect of a clinical trial QA program. NCI and EORTC radiation therapy trials now require centers to participate in regular external dosimetry audits, but in addition to this, most QA programs also require the clinical trial center to demonstrate they have achieved a defined level of accuracy using their in-house equipment for other aspects of the treatment delivery chain.

In addition to treatment delivery accuracy, the QA program must be designed to confirm that all aspects of the protocol were followed. The process for achieving this and the frequency of monitoring protocol compliance varies between trials and is again related to resources and risk. In the ideal world, all treatment plans for all trials would be reviewed prior to patients starting treatment. This is indeed necessary for trials where protocol non-compliance is likely and such non-compliance is likely to impact on the trial outcome. In such situations, it is necessary to support the trial with a team of appropriately qualified reviewers. These reviewers may need to develop plan review consensus guidelines to ensure consistency.

Prior to entering patients into a clinical trial, centers may be required to complete a credentialing program. Details of credentialing programs are discussed later. The advantage of a credentialing program is that centers have the opportunity to demonstrate they have read, understood and are able to follow the trial protocol, and that they are able to submit data to the clinical trials center for review. It also provides the PI with an insight into the likely frequency of protocol violations, identifies ambiguities in the protocol, and assists centers to achieve the objectives of the trial protocol. Some aspects of credentialing can be completed in advance, which can enable centers to be prepared by the time a trial opens.

In addition to credentialing a treating center for clinical trial participation, the basic QA program for most clinical trials involving radiotherapy will require the center to submit a minimum number of treatment plans for review. Clinical trials that permit complex or novel techniques, or require a specific level of treatment delivery accuracy may also incorporate specific QA programs. For example, to use IMRT techniques in an RTOG trial, centers must successfully complete the IMRT phantom exercise (31). Further tests may be required for specific trials. Similar QA programs exist for trials involving IMRT with intrathoracic lesions, image-guided radiation therapy (IGRT), and brachytherapy.

## Credentialing for Radiation Therapy Clinical Trials

A clinical trial may incorporate a credentialing program in an effort to minimize the number of protocol violations and improve the overall quality of the trial (32). Credentialing offers the opportunity to confirm investigators have read and understood the trial protocol and have the necessary resources to treat the patients in all arms of the trial in accordance with the trial’s specifications. When trials allow the introduction of a new technique, credentialing is also an opportunity to provide education, peer review, and an external formal assessment that the new technique has been appropriately commissioned prior to clinical implementation. For example, the UK trial *PARSPORT* allowed many centers to implement IMRT for head-and-neck cancers as a result of participation in the trial (33). Human error has been identified as one of the major contributing factors in patient treatments and therefore some clinical trial groups will require process maps to be developed and submitted for approval prior to a center opening a clinical trial (34).

Credentialing studies may be costly to execute and are often seen as an additional burden in the busy clinic, as it is perceived that no patient will benefit from the exercise. A good credentialing program however, will identify and correct trial protocol ambiguities, provide education to the staff and potentially limit the number of treatment plans that must be re-submitted due to protocol violations. The credentialing program will also encourage improvements in treatment delivery that will affect all patients treated at the center, not just those treated on clinical trials.

### Trial-specific credentialing

Pre-trial credentialing may require centers to demonstrate that they are able to achieve a high level of treatment accuracy which may be an important element in dose escalation studies (35) or studies involving tight treatment margins or hypofractionated schedule (36).

Clinical trials that require the use of advanced technologies such as IMRT and prostate brachytherapy are considered sufficiently challenging that institutions are required to demonstrate their ability to use these technologies before being permitted to register patients (37–39). The RPC participates in the credentialing process for a number of clinical trials through several NCI-funded cooperative groups. In most cases, the RPC collaborates with one or more of several other NCI-funded QA offices, and has on occasion collaborated with international QA offices.

Credentialing for such clinical trials generally involves an evaluation of most if not all of the following aspects:

- Attestation to use of the particular advanced technology to treat patients previously: institutions must demonstrate that they are familiar with the technique and have used it to treat at least some minimum number of patients.
- Facility questionnaire: this questionnaire asks institutions to describe relevant aspects of their treatment planning and delivery equipment, their QA procedures, and in some cases, the personnel who will be participating in protocol patient treatments.
- Knowledge assessment questionnaire: the physician is asked to take a simple quiz to indicate that he or she is familiar with the protocol and its requirements.
- Benchmark case or phantom: for the more complex technologies, the institution may be required to submit a treatment plan generated for a standardized geometry or CT data set (a “dummy run”), or simulate, plan, and treat a geometric or anthropomorphic phantom (an “end-to-end test”). If the protocol requires a benchmark treatment plan, some QA offices such as the RPC review the institution’s plan and re-calculate the doses at key locations to evaluate the accuracy of the planning system. This calculation is possible because the RPC has either measured data collected through visits to the institution, or is able to use the “standard data” described earlier (40). When anthropomorphic phantoms are used, the delivered dose must be compared with the institution’s plan to determine the agreement (1–3, 41–43). An audit of institutions participating in trials run by the TROG was reported by Kron et al. (38). A review of the RPC results was recently reported by Molineu et al. describing more than 1,100 irradiations of a head-and-neck phantom (3).
- Patient-specific plan QA review: as part of credentialing, some trials may require the treating site to also submit their standard in-house dosimetry QA report for the plan created for the treatment planning exercise, for example in the case of IMRT plans. The QA report along with the in-house QA protocol may need to be approved prior to trial activation. In this case the trial medical physicist may be required to assess the suitability of the equipment used by the trial center to perform the QA, the tolerance limits defined in the in-house protocol and the experience of the physics staff in carrying out the QA tasks.
- Electronic data submission: many clinical trials groups require institutions to submit the treatment plans for protocol patients digitally. Several QA offices can receive data electronically, and the Image-guided Therapy QA Center (ITC) was established to assist QA offices and study groups in this regard. The plans performed for irradiation of the anthropomorphic phantoms also must be submitted digitally to facilitate comparison with the institutions’ own treatment plans.
- Quality assurance and dosimetry review: some QA offices, including the RPC, review QA and dosimetry procedures, and records from the participating institutions, to ascertain compliance with published recommendations such as those from the AAPM (44, 45).
- Clinical review by radiation oncologist: in some cases, the protocol requires that the institution submit representative treatment plans performed for patients treated previously using the technology being tested by the protocol, and techniques at least similar to those required by the protocol. The plans are reviewed by the study chair or a radiation oncologist to ensure that they conform to the intentions of the study chair or his/her designee.
- Reviews of patient treatment records: in some cases, a QA office will review the treatment plans prepared by participating institutions for patients registered on a clinical trial. When the protocol is complex and treatment delivery errors could mask the results of the trial, the study chair may require that institutions submit their treatment plans for review before the patients are treated. Such “rapid reviews” ensure that the treatment plans meet the dosimetric requirements of the protocol. An example of a recent protocol that requires rapid reviews is the joint NSABP B-39/RTOG 0413 trial of accelerated partial-breast irradiation (46).

## External Dosimetry Audits

### Remote audits of treatment machine output

At least five organizations conduct regular independent audits of treatment machine output calibration with mailed dosimeters. They include the RPC based in Houston, TX, USA; Radiation Dosimetry Services, also located in Houston; the International Atomic Energy Agency (IAEA) based in Vienna; the European Quality Assurance Laboratory/European Society for Therapeutic Radiology and Oncology (EQUAL-ESTRO) located in Paris; and the Section of Outreach Radiation Oncology and Physics at the National Cancer Center of Japan, in Tokyo. Of these five centers, the RPC has the largest program and monitors all of the institutions (>1,800 institutions as of early 2012) that participate in NCI sponsored clinical trials, both within the USA and internationally (1, 32). The RPC initiated its TLD program for photon beams in 1977 (47, 48). In 1982 electron beams were included, and in 2007, measurements of proton beams were initiated. In 2010, the RPC adopted the use of optically stimulated luminescence dosimeters (OSLDs) and largely discontinued the use of TLDs for photons and electrons (49).

Most external audit systems are relatively simple; the RPC’s system is an example. Each year, institutions receive a package containing a lightweight platform and acrylic mini-phantoms containing several OSLD “nanoDots” (Landauer, Inc., Glenwood, IL, USA) for irradiation with each radiation beam. Instructions are enclosed that explain the irradiation procedure and ask the institution to describe their calibration procedure. The blocks and other equipment are returned to the RPC where the OSLDs are analyzed. The RPC applies corrections for the differences in scatter between the institution’s calibration conditions and the OSLD irradiation, and for fading, dose linearity, and energy dependence of the OSLD system (47).

The uncertainty of the OSLD system to measure output of accelerators remotely has been evaluated and found to be 1.5% (50). This uncertainty is expressed as the standard deviation of measurements of dose with the RPC’s OSLD system. Consequently, the RPC’s measurement of an institution’s output can be stated at an uncertainty of <5% using a 99% confidence interval. The RPC has established ±5% as a threshold for acceptability. When the OSLD measurement disagrees with an institution’s stated dose by more than 5%, the RPC initiates a series of activities to resolve the discrepancy. If the discrepancy cannot be resolved through telephone calls and the review of procedures and documentation, an on-site dosimetry visit is scheduled.

Audits of machine calibration have been described by several other investigators. For example, Rassiah et al. described an independent audit of treatment centers in Malaysia using TLDs (51). Williams et al. described the early experience of the Australian dosimetry audits using TLDs and OSLDs (23). Similarly, Mizuno et al. described an audit system using rods made of a thermoluminescent glass (52). Their study demonstrated that the system was suitable for measurements of treatment machine output, and is proposed for use at a number of treatment centers in Japan. A similar analysis in Korea led to the same conclusion (53). However, the experience at the IAEA in Vienna is comparable to that of the RPC in terms of numbers of separate institutions audited, although the frequency of audits is lower (54).

### Results of remote audits of machine calibration

**Figure 1:**
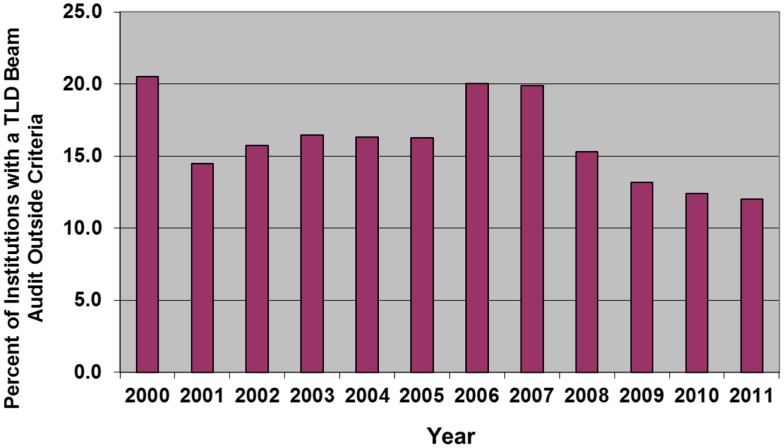
**The percent of institutions irradiating TLD in any year that had at least one beam that failed the RPC’s 5%/5 mm criteria for acceptability**.

The RPC has described results from a large series of annual calibration audits (8, 55). Consistently each year, 5–6% of the US megavoltage beams audited with TLD have fallen outside of the RPC’s ±5% dose or 5 mm electron depth dose criteria on the first measurement. The analysis indicated that the incorrectly calibrated beams are distributed among approximately 10–20% of the institutions monitored by the RPC (Figure 1). This observation has been confirmed by on-site visit measurements using ion chambers; among institutions visited by the RPC, approximately 10–15% had one or more beams outside of the RPC’s criteria on an annual basis that required an investigation by the RPC (8, 55).

The precedent for performing the TLD audit annually was established by the RPC many years ago and all current trial data and results are based on having this level of QA. A review of EORTC trial results indicated that decreases in tumor control probability were associated with discrepancies in the beam calibration, as measured by a TLD audit program (20). At the same time, increases in normal tissue morbidity were associated with discrepant high TLD measurements. The article also indicated that sequential TLD audits improved the uniformity of the clinical outcome and that small deviations in beam output might lead to clinically important variations in outcome. Mailed TLD audits were deemed to be an integral part of QA for trials. Other studies have shown the importance of independent audits to assure clinical trial quality (11, 21).

The RPC data also showed that 41% of the institutions monitored by the RPC had exactly one discrepancy detected by the TLD program during the 10-years between 1998 and 2008 (55, 56). However, thanks at least in part to the RPC’s intervention, a much smaller percentage had two or more discrepancies during this period. As was indicated above, while calibration discrepancies were detected for 15–20% of the major contributors to clinical trials in any 1 year, these institutions did not consistently have such discrepancies. Instead, significant calibration errors apparently can occur at any institution at any time. Approximately 230 new machines are installed each year at institutions participating in US NCI-funded clinical trials. New machines are subject to calibration errors as they are put into clinical service, with potentially serious results. These errors can occur as a result of changes in procedures, the recruiting of inexperienced personnel, and frequently, with the installation of new treatment equipment.

### On-site dosimetry review visits

A dosimetry review audit has been recommended by several organizations, including the AAPM and the IAEA (57, 58). An independent on-site audit is especially important for solo practitioners but is a valuable exercise for all practicing clinical medical physicists. It need not be extensive, but should address key activities such as basic calibrations, the overall QA program and documentation.

A independent dosimetry review visit consists of a review of the institution’s QA procedures and documentation; a review of treatment records to ascertain the consistency of the procedures used for treatment planning and monitor unit calculations; and measurements of the radiation beams and radioactive sources. The measurements should include mechanical alignment and accuracy of position readout devices, light versus radiation field congruency, calibration of treatment machine output and brachytherapy source strength, relative field-size dependence, percent depth dose, off-axis ratio, asymmetric jaw and irregular field parameters, and accessory transmission factors. Additional measurements should evaluate the basic data required for delivery of IMRT, including small-field output factors, and the performance of a multi-leaf collimator (MLC). A straight-forward spot-check of IGRT can be performed with a simple phantom (36). Because of interest in treating protocol patients with protons, the RPC and other QA groups have developed and implemented procedures for visits to proton-beam facilities (59).

The RPC has conducted on-site audits during its 45-year history, and has accumulated extensive measured data from several thousand photon beams which have been grouped into 96 combinations of manufacturer, model, and beam energy (40). This database of “Standard Data” enables the RPC to provide assistance by comparing an institution’s measured data with the Standard Data. Differences often point to measurement errors and help identify the source of calibration errors detected by a mailed audit.

### Measurements of beam parameters during dosimetry review visits

During a dosimetry review visit, a QA office can evaluate many aspects of performance of the institution’s dosimetry system. In the RPC’s case, a report is prepared that indicates measurements that disagree with the institution’s treatment planning dosimetry data, and when the disagreements exceed appropriate thresholds, the report includes recommendations to the institution for improvement. These recommendations then demonstrate areas that require attention by the institution. When considered in aggregate, these recommendations form an indication of the areas of general concern at the visited institutions. The common recommendations and the frequency with which institutions receive them are shown in Table 2 (8). The recommendations indicated by asterisks are considered important dosimetry parameters. On average, 70% of visited institutions received one or more of these recommendations.

**Table 2:** **Some of the discrepancies detected during RPC dosimetry review visits to 156 institutions in 2005–2011**.

Errors regarding	Number of institutions (%)
Inadequacy of QA program	115 (74)
Photon field-size dependence (small fields)*	62 (40)
Wedge transmission factor*	50 (32)
Off-axis factors, beam symmetry	46 (29)
Electron calibration*	27 (17)
Photon depth dose*	25 (16)
Electron depth dose*	18 (11)
Photon calibration*	13 (8)

The parameters indicated by “*” are considered significant dosimetry parameters that influence the calculation of patient treatment meter settings. During this 6-year period, 70% of the visited institutions received one or more recommendations to address discrepancies in these dosimetry parameters.

### Reviews of QA programs

Recommendations for performing routine QA in radiation therapy departments are presently in flux. The only widely accepted published recommendations today are contained in the AAPM’s reports from its Task Groups 40 and 142 on comprehensive QA (44, 45). Consequently, the RPC judges the quality of an institution’s QA program against the AAPM’s TG-40 and -142 recommendations. Significant differences with the AAPM recommendations are found frequently, but in some cases, these are justified by the institution’s own procedures and measurements. More often, however, the institution has overlooked some component of recommended QA, or has allowed their program to lapse in some important aspect. A list of common failures or lapses in QA programs found by the RPC appears in Table 3 (8).

**Table 3:** **Common QA lapses and deficiencies found at institutions during RPC visits**.

QA records not available or maintained
Annual calibrations or monthly checks not performed timely
No record of comparison to clinical values on annual report
No record of comparison of daily and monthly checks against annual “baseline” values
Physicist review of daily checks not documented
No record of corrective actions and repeat measurements
Check of electron beam energy not performed as required by applicable regulations and recommendations
Output or field flatness constancy with gantry angle not checked during annual calibration

### Observations from reviews of patient records

Quality assurance offices often participate in the QA review of the treatment records of patients treated on protocols managed by cooperative study groups. The QA office’s role can vary with the trial, but in the RPC’s case, this role often incorporates an independent recalculation of the patient dose, and a comparison with both the dose stated by the institution and the dose required by the protocol. The independent calculation is based on the treatment parameters stated by the institution, such as field size and MU setting, but utilizes the RPC’s data from the institution’s treatment machines. If the RPC has conducted a dosimetry review visit to the institution, the measured beam parameters are used for calculation. If the RPC has not visited, the standard data (40) are used together with measurements of machine calibration from the RPC’s annual TLD audits.

Results from 5 years of the RPC’s reviews of charts were analyzed in preparation for submission of a grant renewal application in 2010. The data were collected from 2004 to 2008 and included 1,506 patients for which doses were calculated at 8,448 points. The discrepancies were divided into systematic errors, individual errors, and transcription errors. To be identified as an error, the patient dose had to disagree with the institution’s stated dose by more than 5% for external beam treatment, and by more than 15% for brachytherapy treatments. Systematic errors were those believed to affect all patients at an institution, who were treated with a specific treatment machine or source, or for which a particular device such as a wedge, was used. Such errors were relatively infrequent, but were found in 1% of the records reviewed. Individual errors were those believed to affect only the patient in question. These errors occurred more frequently, about 11% of the time. Transcription errors reflected cases in which the data reported to the study group did not accurately reflect data recorded in the treatment record. Such errors occurred surprisingly frequently; about 27% of the time.

All together, 39% of the charts reviewed by the RPC contained one or more of the errors described above. In each case, the error was corrected by the RPC and reported to the study group so that correct information could be used for evaluation of the clinical trial. The results of these reviews were also reported to the institutions promptly, to enable the institutions to take corrective action. When errors affecting patient dose were detected, the RPC contacted the institution to confirm that the records accurately reflected the patient’s treatment, and that the RPC understood the institution’s calculation procedures. When the errors were confirmed, the RPC conveyed the details of its calculations and investigated the reasons for the discrepancy.

### Results of treatment planning benchmark tests

For some trials, several QA offices have developed techniques to credential institutions through the use of a treatment planning exercise called a “benchmark case.” The purpose of the benchmark case is to test whether an institution can meet one or more requirements specified in the trial, such as dose prescription, contouring, etc. To comply, an institution must download a standardized CT data set (or comparable imaging information) and generate a treatment plan that complied with the requirements of the relevant trial. The treatment plan must then be submitted digitally for evaluation. Evaluation generally consisted of review of target volume contours and DVHs (60, 61). However, when used by the RPC, an independent calculation of dose to the target was customarily performed. When benchmark cases failed to meet the criteria, the RPC contacted the institution, explained the discrepancies, and worked with the institution to resolve them. The follow-up generally consisted of irradiation of an anthropomorphic phantom as a more definitive end-to-end test of the treatment planning capability.

### Results of anthropomorphic phantom reviews

Several QA offices have reported the results of independent audits with an anthropomorphic phantom. During the time period 2001–2011 the RPC mailed head-and-neck phantoms to 763 distinct institutions. The institutions were instructed to perform imaging, develop a treatment plan using IMRT techniques, and then deliver the treatment to the phantom. A total of 1,139 irradiations were analyzed. Of these, 929 irradiations or 82% successfully met the irradiation criteria of 7% and 4 mm distance-to-agreement (DTA). The passing rate has increased steadily since the phantom’s introduction in 2001 from approximately 69% to a current rate of 91% (3).

Had the dose criterion been reduced to 5%, only 69% of the irradiations would have passed. Two hundred and ten irradiations failed to meet the irradiation criteria; of these the majority (74%) failed only the dose criterion. The remaining unsuccessful irradiations failed the DTA criterion or both the dose and DTA criteria. Some institutions have irradiated a phantom, particularly the RPC’s head-and-neck phantom, multiple times because they wanted to improve their initial irradiation results, test different treatment planning system algorithms or test different treatment delivery systems.

A study was performed comparing the results from the benchmark case used by the Quality Assurance Review Center as an IMRT treatment planning capability exercise, to the RPC IMRT H&N phantom irradiation results from the same group of 113 institutions was performed. All of the institutions passed the IMRT benchmark case (planning study and self-reported IMRT QA measurement) while 20 of these same institutions did not pass the end-to-end independent phantom irradiation. The phantom audit is capable of detecting imaging, data transfer and delivery errors that cannot be detected by the institution’s completion of a benchmark case. In addition, each institution’s own IMRT QA results of their IMRT benchmark plans passed their own QA criteria. An independent end-to-end anthropomorphic QA audit such as those used by the RPC are capable of detecting dosimetry errors that might otherwise go undetected.

The most common TPSs used to plan the irradiations of the phantom were the Phillips Pinnacle and Varian Eclipse systems. The pass rates for these two TPSs were approximately 75 and 88%, respectively. The difference is believed to be due to difficulties in modeling the penumbra at the ends of rounded MLC leaves (62).

## Incorporating New and Complex Technologies into Clinical Trials

Clinical trials that are open to recruitment over a protracted period typically struggle to keep pace with new technology as it is implemented in the clinic. There needs to be a balance between ensuring the new technology will not impact on the ability of the trial to answer the trial question, but at the same time, it is important that trials produce data using treatment techniques that are not outdated. The CCTGs may try to anticipate upcoming developments, but it is often difficult to develop a QA and credentialing program in a rapid time span due to limited access to equipment, limited experience of the trial teams, and lack of published data to provide the evidence to support the introduction of the new technology (1, 29).

New trials that deliberately set-out to incorporate new technology offer an ideal opportunity for medical physicists to exchange knowledge and experience as the new technology is introduced into the clinic. Credentialing programs are designed to assist centers to meet protocol requirements. The results of all studies within a clinical trial remain anonymous, and a good credentialing team will offer support to those centers that struggle to meet the goals of the protocol. This in turn offers benefits to patients who can be assured that the new technology has undergone peer review prior to clinical implementation. It is important to note however, that it is the responsibility of the hospital staff to ensure patient safety, and participating in a clinical trial credentialing program can support, but not take on that role.

In designing a clinical trial that incorporates new technology, the clinical trial management committee must first agree on the level of support that it is able to offer radiotherapy centers. Trials running on a limited budget or with minimal support may exclude centers without a significant amount of experience in using the new technology so that only limited support is required. This may introduce bias into the clinical trial results as the results may only achievable in highly experienced centers. Ideally, a trial that permits a new technology should set up a comprehensive credentialing program including site visits, a detailed QA program, and set up a network for providing support for centers with similar equipment such as the comprehensive QA program conducted during the introduction of 3DCRT in UK centers participating in the MRC RT01 trial (34, 63–65).

## Conclusion

Clinical trials can be a powerful tool for demonstrating the effectiveness of a clinical intervention or treatment. In radiation therapy, clinical trials have proven the benefits (or lack thereof) of specific fractionation regimens, advantages of combinations of radiation with chemotherapeutic agents and radiation sensitizers, and the potential of new modalities. However, the most compelling trials are those in which the benefits of the proposed treatment methodology are evaluated through multicenter Phase III randomized trials. While such trials can potentially demonstrate the ability of the community at large to deliver the treatment effectively and thus reap the benefits, these trials also are more likely to suffer from variations in treatment quality among the participating institutions. A comprehensive QA program can define the range of acceptable and unacceptable variations, detect, and correct the causes of such variations, and document the frequency of variations. The ability of QA programs to reduce variations in treatment delivery and improve the quality of clinical trials has been demonstrated. For such QA programs to be effective, the clinical trials cooperative groups as well as the participating institutions must embrace them. Education in clinical trials QA should be offered regularly at venues such as national and international professional and scientific meetings, and at meetings of the cooperative groups themselves.

## Conflict of Interest Statement

The authors declare that the research was conducted in the absence of any commercial or financial relationships that could be construed as a potential conflict of interest.

## References

[1] Ibbott GS Followill DS Molineu HA Lowenstein JR Alvarez PE Roll JE Challenges in credentialing institutions and participants in advanced technology multi-institutional clinical trials. Int J Radiat Oncol Biol Phys (2008) 71(1 Suppl):S71–5 10.1016/j.ijrobp.2007.08.083 18406942 PMC2409281

[2] Molineu A Followill DS Balter PA Hanson WF Gillin MT Huq MS Design and implementation of an anthropomorphic quality assurance phantom for intensity-modulated radiation therapy for the Radiation Therapy Oncology Group. Int J Radiat Oncol Biol Phys (2005) 63(2):577–83 10.1016/j.ijrobp.2005.05.021 16168849

[3] Molineu A Hernandez N Nguyen T Ibbott G Followill D Credentialing results from IMRT irradiations of an anthropomorphic head and neck phantom. Med Phys (2013) 40(2):022101 10.1118/1.4773309 23387762 PMC3555917

[4] Ibbott GS Hanson WF O’Meara E Kuske RR Arthur D Rabinovitch R Dose specification and quality assurance of Radiation Therapy Oncology Group protocol 95-17; a cooperative group study of iridium-192 breast implants as sole therapy. Int J Radiat Oncol Biol Phys (2007) 69(5):1572–8 10.1016/j.ijrobp.2007.08.011 18035213 PMC2180157

[5] CEBM Centre for Evidence-Based Medicine (2011). Available from: http://www.cebm.net

[6] Haworth A Ibbott G Medical physics for clinical trials. In: Van Dyke J, editor. The Modern Technology of Radiation Oncology: A Compendium for Medical Physicists and Radiation Oncologists. Madison, WI: Medical Physics Publishing (2013). p. 487–510

[7] Olch AJ, editor. *Quality Assurance for Clinical Trials: A Primer for Physicists* AAPM reports. Madison, WI: Medical Physics Publishing (2004). Report No.: 86.

[8] Ibbott GS QA in radiation therapy: the RPC perspective. J Phys (2010) 250:1–7 10.1088/1742-6596/250/1/012001

[9] Kernan WN Viscoli CM Makuch RW Brass LM Horwitz RI Stratified randomization for clinical trials. J Clin Epidemiol (1999) 52(1):19–26 10.1016/S0895-4356(98)00138-3 9973070

[10] Kaplan RS How can we improve clinical trials? Nat Clin Pract Oncol (2007) 4(4):206–7 10.1038/ncponc0775 17392712

[11] Pettersen MN Aird E Olsen DR Quality assurance of dosimetry and the impact on sample size in randomized clinical trials. Radiother Oncol (2008) 86(2):195–9 10.1016/j.radonc.2007.07.001 17727987

[12] ICRU Prescribing, Recording and Reporting Photon Beam Therapy (Report 50). Bethesda, MD: International Commission on Radiation Units and Measurement (ICRU) (1993).

[13] ICRU Prescribing, Recording and Reporting Photon Beam Therapy (Supplement to ICRU Report 50). Bethesda, MD: International Commission on Radiation Units and Measurement (ICRU) (1999).

[14] ICRU Prescribing, Recording, and Reporting Intensity-Modulated Photon-Beam Therapy (IMRT) (ICRU Report 83). Bethesda, MD: International Commission on Radiation Units and Measurement (ICRU) (2010).

[15] Das IJ Cheng CW Chopra KL Mitra RK Srivastava SP Glatstein E Intensity-modulated radiation therapy dose prescription, recording, and delivery: patterns of variability among institutions and treatment planning systems. J Natl Cancer Inst (2008) 100(5):300–7 10.1093/jnci/djn020 18314476

[16] Willins J Kachnic L Clinically relevant standards for intensity-modulated radiation therapy dose prescription. J Natl Cancer Inst (2008) 100(5):288–90 10.1093/jnci/djn039 18314470

[17] Kry SF Alvarez P Molineu A Amador C Galvin J Followill DS Algorithms used in heterogeneous dose calculations show systematic differences as measured with the Radiological Physics Center’s anthropomorphic thorax phantom used for RTOG credentialing. Int J Radiat Oncol Biol Phys (2013) 85(1):e95–100 10.1016/j.ijrobp.2012.08.039 23237006 PMC3522855

[18] Marks LB Ten Haken RK Martel MK Guest editor’s introduction to QUANTEC: a users guide. Int J Radiat Oncol Biol Phys (2010) 76(3 Suppl):S1–2 10.1016/j.ijrobp.2009.08.075 20171501

[19] Law MY Liu B Chan LW Informatics in radiology: DICOM-RT-based electronic patient record information system for radiation therapy. Radiographics (2009) 29(4):961–72 10.1148/rg.294085073 19448106

[20] Bentzen SM Bernier J Davis JB Horiot JC Garavaglia G Chavaudra J Clinical impact of dosimetry quality assurance programmes assessed by radiobiological modelling of data from the thermoluminescent dosimetry study of the European Organization for Research and Treatment of Cancer. Eur J Cancer (2000) 36(5):615–20 10.1016/S0959-8049(99)00336-6 10738126

[21] Peters LJ O’Sullivan B Giralt J Fitzgerald TJ Trotti A Bernier J Critical impact of radiotherapy protocol compliance and quality in the treatment of advanced head and neck cancer: results from TROG 02.02. J Clin Oncol (2010) 28(18):2996–3001 10.1200/JCO.2009.27.4498 20479390

[22] van Tienhoven G Mijnheer BJ Bartelink H Gonzalez DG Quality assurance of the EORTC Trial 22881/10882: boost versus no boost in breast conserving therapy. An overview. Strahlenther Onkol (1997) 173(4):201–7 10.1007/BF03039289 9111608

[23] Williams I Kenny J Lye J Lehmann J Dunn L Kron T The Australian Clinical Dosimetry Service: a commentary on the first 18 months. Australas Phys Eng Sci Med (2012) 35(4):407–11 10.1007/s13246-012-0161-1 23055126 PMC3562435

[24] Reinstein LE Peachey S Laurie F Glicksman AS Impact of a dosimetry review program on radiotherapy in group trials. Int J Radiat Oncol Biol Phys (1985) 11(6):1179–84 10.1016/0360-3016(85)90067-7 3888935

[25] Glicksman AS Reinstein LE Laurie F Quality assurance of radiotherapy in clinical trials. Cancer Treat Rep (1985) 69(10):1199–205 4042099

[26] Ohri N Shen X Dicker AP Doyle LA Harrison AS Showalter TN Radiotherapy protocol deviations and clinical outcomes: a meta-analysis of cooperative group clinical trials. J Natl Cancer Inst (2013) 105(6):387–93 10.1093/jnci/djt001 23468460 PMC3601950

[27] Fairchild A Straube W Laurie F Followill D Does quality of radiation therapy predict outcomes of multicenter cooperative group trials? A literature review. Int J Radiat Oncol Biol Phys (2013) 87(2):246–60 10.1016/j.ijrobp.2013.03.036 23683829 PMC3749289

[28] Rischin D Peters LJ O’Sullivan B Giralt J Fisher R Yuen K Tirapazamine, cisplatin, and radiation versus cisplatin and radiation for advanced squamous cell carcinoma of the head and neck (TROG 02.02, HeadSTART): a phase III trial of the Trans-Tasman Radiation Oncology Group. J Clin Oncol (2010) 28(18):2989–95 10.1200/JCO.2009.27.4449 20479425

[29] Purdy JA Quality assurance issues in conducting multi-institutional advanced technology clinical trials. Int J Radiat Oncol Biol Phys (2008) 71(Suppl):S66–70 10.1016/j.ijrobp.2007.07.2393 18406941

[30] Pawlicki T Whitaker M Boyer AL Statistical process control for radiotherapy quality assurance. Med Phys (2005) 32(9):2777–86 10.1118/1.2001209 16266091

[31] RPC (2011). Available from: http://rpc.mdanderson.org/rpc/credentialing/IMRTbenchmark-021704.pdf

[32] Followill DS Urie M Galvin JM Ulin K Xiao Y Fitzgerald TJ Credentialing for participation in clinical trials. Front Oncol (2012) 2:198 10.3389/fonc.2012.00198 23272300 PMC3530078

[33] Clark CH Miles EA Urbano MT Bhide SA Bidmead AM Harrington KJ Pre-trial quality assurance processes for an intensity-modulated radiation therapy (IMRT) trial: PARSPORT, a UK multicentre phase III trial comparing conventional radiotherapy and parotid-sparing IMRT for locally advanced head and neck cancer. Br J Radiol (2009) 82(979):585–94 10.1259/bjr/31966505 19332518

[34] Mayles WP Moore AR Aird EG Bidmead AM Dearnaley DP Griffiths SE Questionnaire based quality assurance for the RT01 trial of dose escalation in conformal radiotherapy for prostate cancer (ISRCTN 47772397). Radiother Oncol (2004) 73(2):199–207 10.1016/j.radonc.2004.08.017 15622611

[35] Haworth A Kearvell R Greer PB Hooton B Denham JW Lamb D Assuring high quality treatment delivery in clinical trials – results from the Trans-Tasman Radiation Oncology Group (TROG) study 03.04 “RADAR” set-up accuracy study. Radiother Oncol (2009) 90(3):299–306 10.1016/j.radonc.2008.10.011 19017549

[36] Middleton M Frantzis J Healy B Jones M Murry R Kron T Successful implementation of image-guided radiation therapy quality assurance in the Trans Tasman Radiation Oncology Group 08.01 PROFIT Study. Int J Radiat Oncol Biol Phys (2011) 81(5):1576–81 10.1016/j.ijrobp.2010.09.017 21074334

[37] Hurkmans CW van Lieshout M Schuring D van Heumen MJ Cuijpers JP Lagerwaard FJ Quality assurance of 4D-CT scan techniques in multicenter phase III trial of surgery versus stereotactic radiotherapy (radiosurgery or surgery for operable early stage (stage 1A) non-small-cell lung cancer [ROSEL] study). Int J Radiat Oncol Biol Phys (2011) 80(3):918–27 10.1016/j.ijrobp.2010.08.017 20950961

[38] Kron T Hamilton C Roff M Denham J Dosimetric intercomparison for two Australasian clinical trials using an anthropomorphic phantom. Int J Radiat Oncol Biol Phys (2002) 52(2):566–79 10.1016/S0360-3016(01)02682-7 11872306

[39] Palta JR Deye JA Ibbott GS Purdy JA Urie MM Credentialing of institutions for IMRT in clinical trials. Int J Radiat Oncol Biol Phys (2004) 59(4):1257–9; author reply 1259–61 10.1016/j.ijrobp.2004.03.007 15234063

[40] Lowenstein J Kry S Molineu A Alvarez P Aguirre J Summers P High energy photon standard dosimetry data: a quality assurance tool. Med Phys (2012) 39:3754 10.1118/1.4735286 28517347

[41] Followill DS Evans DR Cherry C Molineu A Fisher G Hanson WF Design, development, and implementation of the radiological physics center’s pelvis and thorax anthropomorphic quality assurance phantoms. Med Phys (2007) 34(6):2070–6 10.1118/1.2737158 17654910

[42] Ibbott GS Molineu A Followill DS Independent evaluations of IMRT through the use of an anthropomorphic phantom. Technol Cancer Res Treat (2006) 5(5):481–7 16981790 10.1177/153303460600500504

[43] Oldham M Sakhalkar H Adamovics J Molineu A Ibbott G The feasibility of comprehensive IMRT verification using novel 3D dosimetry techniques compatible with the RPC head and neck phantom. Int J Radiat Oncol Biol Phys (2008) 72(1):S145 10.1016/j.ijrobp.2008.06.469

[44] Klein EE Hanley J Bayouth J Yin FF Simon W Dresser S Task Group 142 report: quality assurance of medical accelerators. Med Phys (2009) 36(9):4197–212 10.1118/1.3190392 19810494

[45] Kutcher GJ Coia L Gillin M Hanson WF Leibel S Morton RJ Comprehensive QA for radiation oncology: report of AAPM Radiation Therapy Committee Task Group 40. Med Phys (1994) 21(4):581–618 10.1118/1.597316 8058027

[46] Vicini F Winter K Wong J Pass H Rabinovitch R Chafe S Initial efficacy results of RTOG 0319: three-dimensional conformal radiation therapy (3D-CRT) confined to the region of the lumpectomy cavity for stage I/II breast carcinoma. Int J Radiat Oncol Biol Phys (2010) 77(4):1120–7 10.1016/j.ijrobp.2009.06.067 19910132 PMC3365530

[47] Kirby TH Hanson WF Gastorf RJ Chu CH Shalek RJ Mailable TLD system for photon and electron therapy beams. Int J Radiat Oncol Biol Phys (1986) 12(2):261–5 10.1016/0360-3016(86)90107-0 3949577

[48] Kirby TH Hanson WF Johnston DA Uncertainty analysis of absorbed dose calculations from thermoluminescence dosimeters. Med Phys (1992) 19(6):1427–33 10.1118/1.596797 1461205

[49] Homnick J Ibbott G Springer A Aguirre J Optically stimulated luminescence (OSL) dosimeters can be used for remote dosimetry services. Med Phys (2008) 35:2994 10.1118/1.2962948

[50] Aguirre J Alvarez P Ibbott G Followill D Analysis of uncertainties for the RPC remote dosimetry using optically stimulated light dosimetry (OSDL). Med Phys (2011) 38:3515 10.1118/1.3612077

[51] Rassiah P Ng KH DeWerd LA Kunugi K A thermoluminescent dosimetry postal dose inter-comparison of radiation therapy centres in Malaysia. Australas Phys Eng Sci Med (2004) 27(1):25–9 10.1007/BF03178885 15156705

[52] Mizuno H Kanai T Kusano Y Ko S Ono M Fukumura A Feasibility study of glass dosimeter postal dosimetry audit of high-energy radiotherapy photon beams. Radiother Oncol (2008) 86(2):258–63 10.1016/j.radonc.2007.10.024 18023489

[53] Rah JE Kim S Cheong KH Lee JW Chung JB Shin DO Feasibility study of radiophotoluminescent glass rod dosimeter postal dose intercomparison for high energy photon beam. Appl Radiat Isot (2009) 67(2):324–8 10.1016/j.apradiso.2008.09.018 19038552

[54] Izewska J Andreo P The IAEA/WHO TLD postal programme for radiotherapy hospitals. Radiother Oncol (2000) 54(1):65–72 10.1016/S0167-8140(99)00164-4 10719701

[55] Ibbott GS QA for Clinical Dosimetry, with Emphasis on Clinical Trials, in Clinical Dosimetry Measurements in Radiotherapy. Madison, WI: Medical Physics Publishing (2009).

[56] Ibbott GS Clinical Trials: Credentialing, in Quality and Safety in Radiotherapy. Boca Raton, FL: Taylor and Francis (2011).

[57] Halvorsen PH Das IJ Fraser M Freedman DJ Rice RE III Ibbott GS AAPM Task Group 103 report on peer review in clinical radiation oncology physics. J Appl Clin Med Phys (2005) 6(4):50–64 10.1120/jacmp.2026.25362 16421500 PMC5723459

[58] IAEA Comprehensive Audits of Radiotherapy Practices: A Tool for Quality Improvement – QUATRO. Vienna: International Atomic Energy Agency (2007).

[59] Summers P Ibbott G Moyers M Grant R Followill D The approval process for the use of proton therapy in NCI sponsored clinical trials. Med Phys (2012) 39:3866 10.1118/1.4735785 28518264

[60] Mayo CS Urie MM A systematic benchmark method for analysis and comparison of IMRT treatment planning algorithms. Med Dosim (2003) 28(4):235–42 10.1016/j.meddos.2003.05.002 14684188

[61] Ulin K Urie MM Cherlow JM Results of a multi-institutional benchmark test for cranial CT/MR image registration. Int J Radiat Oncol Biol Phys (2010) 77(5):1584–9 10.1016/j.ijrobp.2009.10.017 20381270 PMC2906611

[62] Cadman P Bassalow R Sidhu NP Ibbott G Nelson A Dosimetric considerations for validation of a sequential IMRT process with a commercial treatment planning system. Phys Med Biol (2002) 47(16):3001–10 10.1088/0031-9155/47/16/314 12222862

[63] Moore AR Warrington AP Aird EG Bidmead AM Dearnaley DP A versatile phantom for quality assurance in the UK Medical Research Council (MRC) RT01 trial (ISRCTN47772397) in conformal radiotherapy for prostate cancer. Radiother Oncol (2006) 80(1):82–5 10.1016/j.radonc.2006.06.003 16828908

[64] Stanley S Griffiths S Sydes MR Moore AR Syndikus I Dearnaley DP Accuracy and reproducibility of conformal radiotherapy using data from a randomised controlled trial of conformal radiotherapy in prostate cancer (MRC RT01, ISRCTN47772397). Clin Oncol (R Coll Radiol) (2008) 20(8):582–90 10.1016/j.clon.2008.04.019 18565744 PMC2568874

[65] Sydes MR Stephens RJ Moore AR Aird EG Bidmead AM Fallowfield LJ Implementing the UK Medical Research Council (MRC) RT01 trial (ISRCTN 47772397): methods and practicalities of a randomised controlled trial of conformal radiotherapy in men with localised prostate cancer. Radiother Oncol (2004) 72(2):199–211 10.1016/j.radonc.2004.04.007 15297138

